# Supplementary material for: A homologue of the Parkinson’s disease-associated protein LRRK2 undergoes a monomer-dimer transition during GTP turnover
Source: Nat Commun. 2017 Oct 18;8:1008. doi: 10.1038/s41467-017-01103-4 (PMC5714945; doi:10.1038/s41467-017-01103-4)
Supplement: Supplementary file 1 — Supplementary Information [file 41467_2017_1103_MOESM1_ESM.pdf]

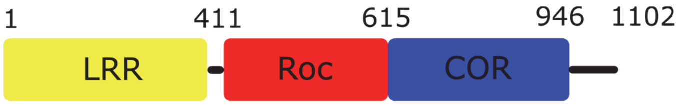

Supplementary Figure 1: Domain organization of the *Chlorobium tepidum* Roco protein.

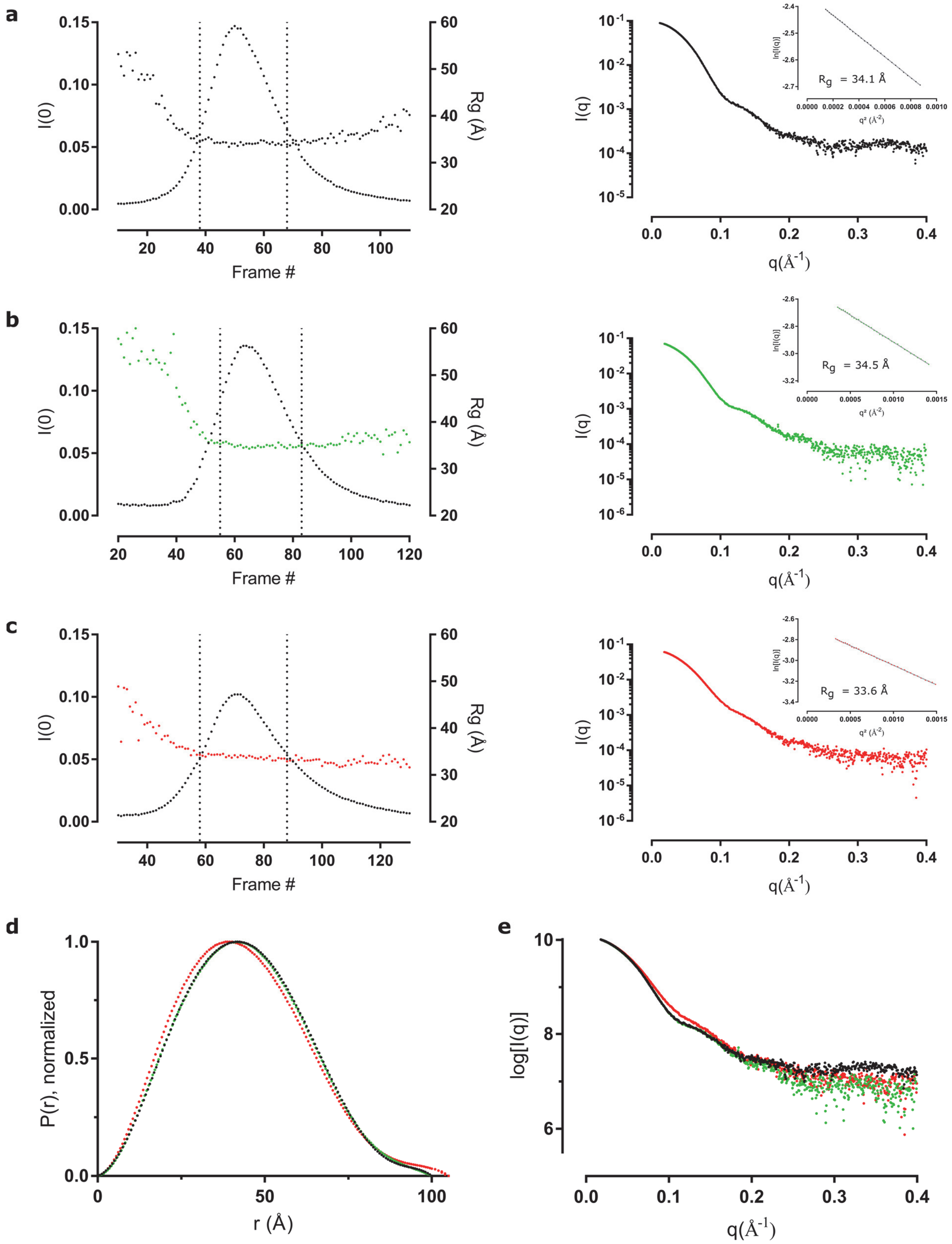
a
I(0)
Rg (Å)
Frame #
I(q)
q(Å-1)
ln[I(q)]
q² (Å-2)
Rg = 34.1 Å
b
I(0)
Rg (Å)
Frame #
I(q)
q(Å-1)
ln[I(q)]
q² (Å-2)
Rg = 34.5 Å
c
I(0)
Rg (Å)
Frame #
I(q)
q(Å-1)
ln[I(q)]
q² (Å-2)
Rg = 33.6 Å
d
P(r), normalized
r (Å)
e
log[I(q)]
q(Å-1)

Supplementary Figure 2: (**a-c**) SEC-SAXS data of CtRoc-COR in nucleotide-free state (**a**, black), bound to GDP (**b**, green) and bound to GppNHp (**c**, red). The chromatograms of the inline size-exclusion chromatography together with the forward scattering (I(0)) and radius of gyration (R_g) obtained from collection of SAXS data on each frame are shown in the left panel. Frames within the dashed lines were selected for averaging. The corresponding final SAXS curves are shown on the right with their Guinier plots as an inset. (**d**) Normalized pair-distance distribution functions (P(r)) of CtRoc-COR in nucleotide-free state (black) or bound to GDP (green) or GppNHp (red). (**e**) Scaled SAXS curves of CtRoc-COR in nucleotide-free state (black), bound to GDP (green) and bound to GppNHp (red).

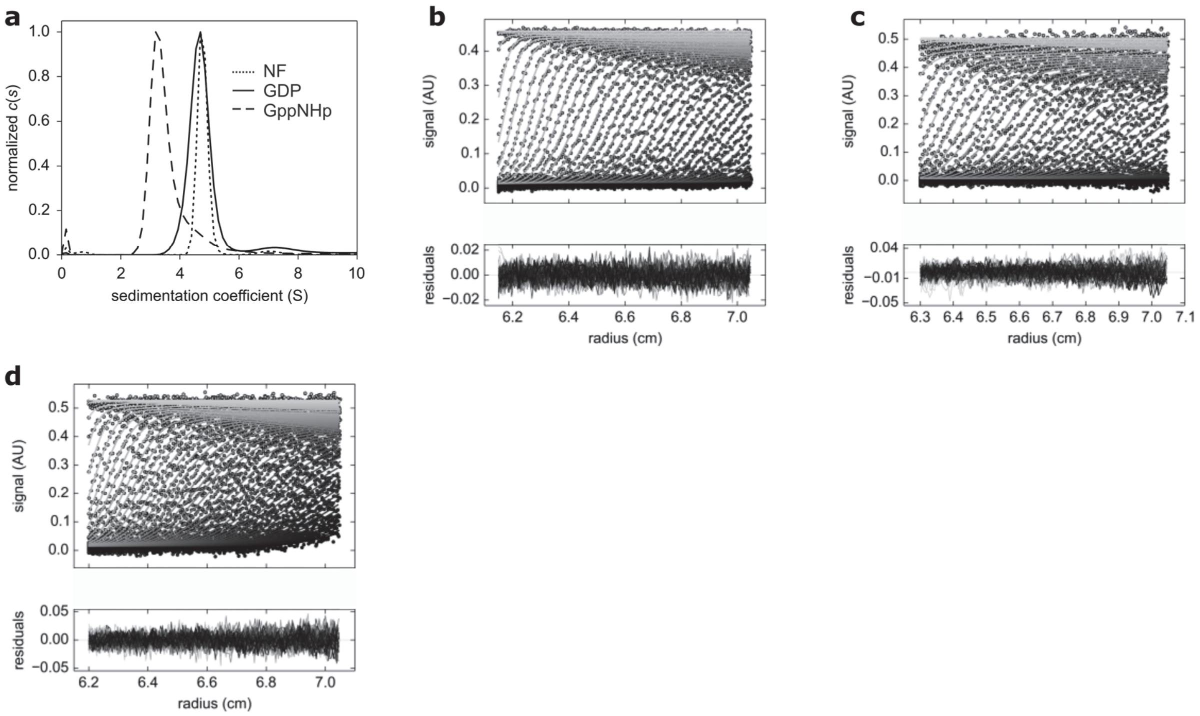

Supplementary Figure 3: Sedimentation velocity analytical ultracentrifugation (SV-AUC) analysis of CtRoc-COR. (**a**) Sedimentation coefficient (*c(s)*) distribution profiles of CtRoc-COR in the presence of 100 µM GppNHp (dashed line, *s* = 3.6 S, estimated mass = 69 kDa, frictional ratio f/f_0 = 1.3), 100 µM GDP (solid line, *s* = 4.7 S, estimated mass = 115 kDa, frictional ratio f/f_0 = 1.4) or absence of nucleotides (dotted line, *s* = 4.8 S, estimated mass = 121 kDa, frictional ratio f/f_0 = 1.4). (**b**-**d**) Fit of the experimental data (dotted line) using the continuous *c(s)* distribution model (solid line) (top) and the residuals (bottom) for (**b**) nucleotide-free CtRoc-COR (rmsd = 0.0063), (**c**) GDP-bound CtRoc-COR (rmsd = 0.011) and (**d**) GppNHp-bound CtRoc-COR (rmsd = 0.012).

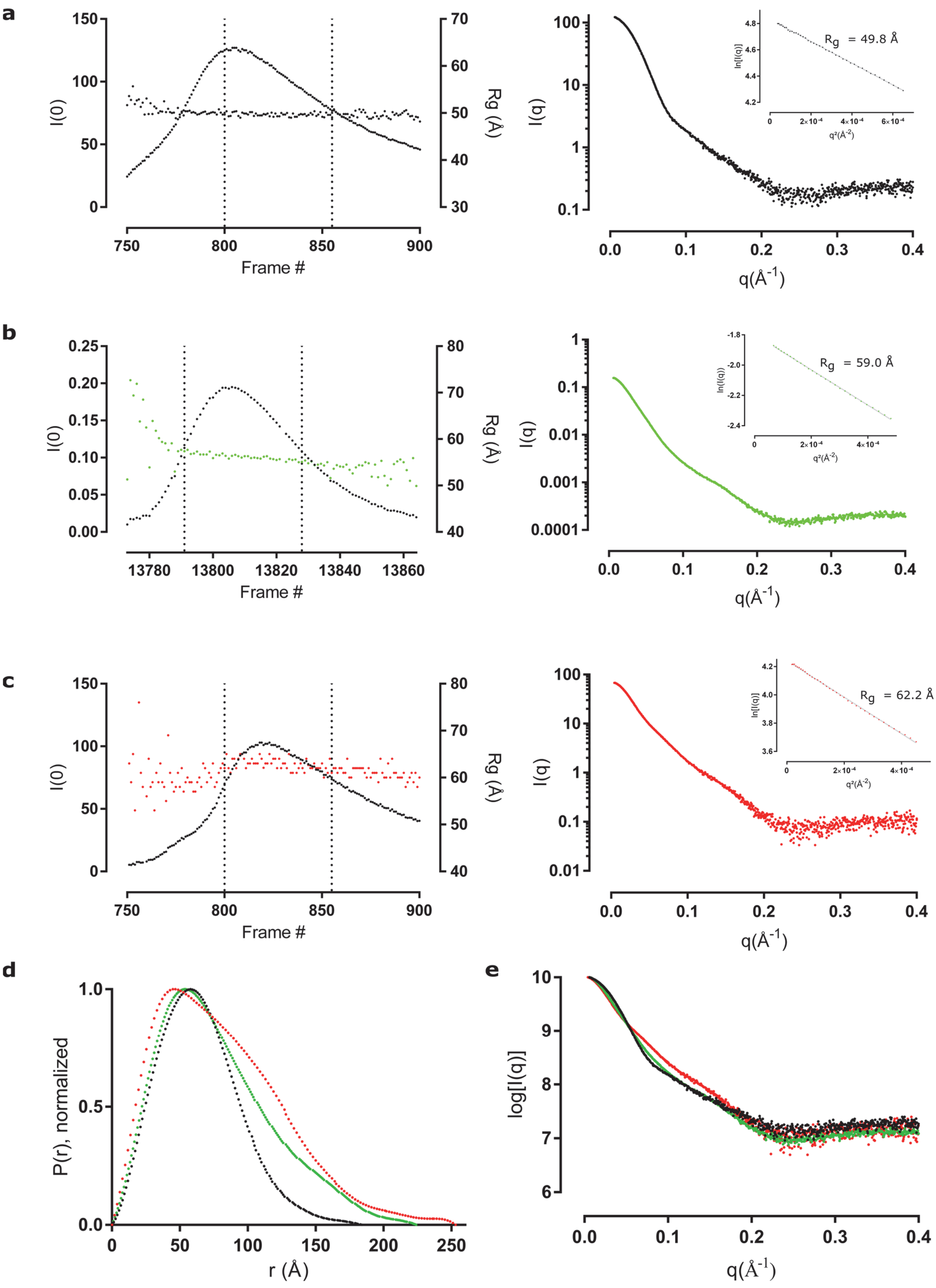
a
I(0)
Rg (Å)
Frame #
I(q)
q(Å-1)
Rg = 49.8 Å
ln[I(q)]
q²(Å-2)
b
I(0)
Rg (Å)
Frame #
I(q)
q(Å-1)
Rg = 59.0 Å
ln(I(q))
q²(Å-2)
c
I(0)
Rg (Å)
Frame #
I(q)
q(Å-1)
Rg = 62.2 Å
ln[I(q)]
q²(Å-2)
d
P(r), normalized
r (Å)
e
log[I(q)]
q(Å-1)

Supplementary Figure 4: (**a-c**) SEC-SAXS data of CtRoco in nucleotide-free state (**a**, black), bound to GDP (**b**, green) and bound to GppNHp (**c**, red). The chromatograms of the inline size-exclusion chromatography together with the forward scattering (I(0)) and radius of gyration (R_g) obtained from collection of SAXS data on each frame are shown in the left panel. Frames within the dashed lines were selected for averaging. The corresponding final SAXS curves are shown on the right with their Guinier plots as an inset. (**d**) Normalized pair-distance distribution functions (P(r)) of CtRoco in nucleotide-free state (black) or bound to GDP (green) or GppNHp (red). (**e**) Scaled SAXS curves of CtRoco in nucleotide-free state (black), bound to GDP (green) and bound to GppNHp (red).

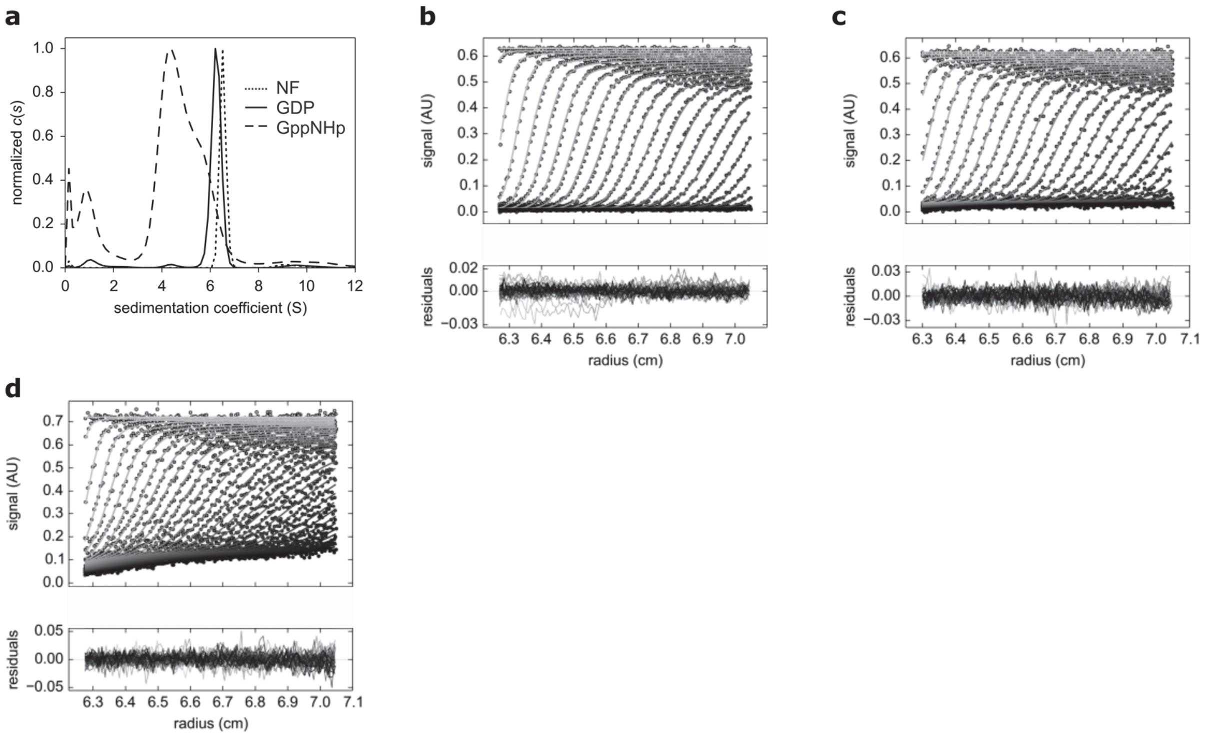

Supplementary Figure 5: Sedimentation velocity analytical ultracentrifugation (SV-AUC) analysis of CtRoco. (**a**) Sedimentation coefficient (*c(s)*) distribution profiles of the CtRoco in the presence of 100 µM GppNHp (dashed line, *s* = 4.9 S, estimated mass = 158 kDa, frictional ratio f/f_0 = 1.91), 100 µM GDP (solid line, *s* = 6.2 S, estimated mass = 239 kDa, frictional ratio f/f_0 =1.96) or absence of nucleotides (dotted line, *s* = 6.5 S, estimated mass = 241 kDa, frictional ratio f/f_0 =1.88). (**b**-**d**) Fit of the experimental data (dotted line) using the continuous *c(s)* distribution model (solid line) (top) and the residuals (bottom) for (**b**) nucleotide-free CtRoco (rmsd = 0.0051), (**c**) GDP-bound CtRoco (rmsd = 0.0078) and (**d**) GppNHp-bound CtRoco (rmsd = 0.011).

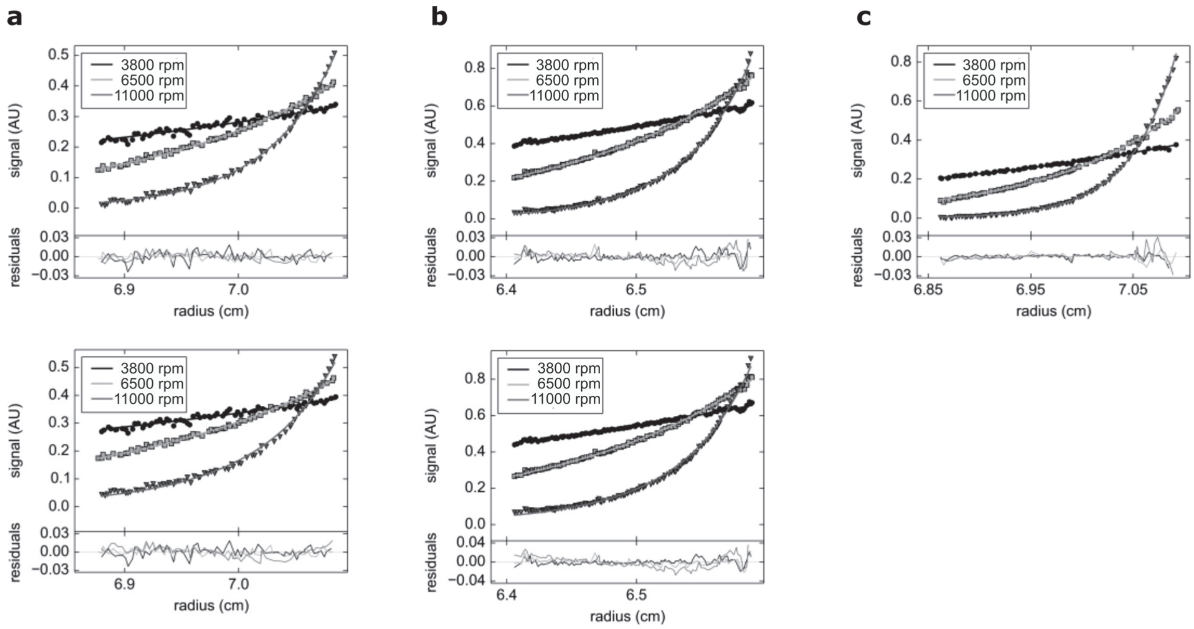

Supplementary Figure 6: Sedimentation equilibrium analytical ultracentrifugation (SE-AUC) analysis of CtRoco. Sedimentation equilibrium AUC data measured at 280 nm for one representative concentration at three different centrifugation speeds of (**a**) GppNHp-bound CtRoco (data for 2.7 µM shown), (**b**) GDP-bound CtRoco (4.5 µM) and (**c**) nucleotide-free CtRoco (2.7 µM) are shown. Top panels are the results from global fitting of 9 datasets (3 concentrations at 3 speeds panel (a)) and 6 datasets (2 concentrations at 3 speeds, panel (b) and (c)) using a single species model, as implemented in the SEDPHAT software. The lower panels (only for GppNHp and GDP) are the global fitting results using a monomer-dimer model. Global reduced Chi² values for data fitting are 2.4 (a, top: GppNHp, single species model), 3 (a, bottom: GppNHp monomer-dimer model), 3.7 (b, top: GDP, single species model), 3.8 (b, bottom: GDP monomer-dimer model), 1.5 (c: NF, single species model).

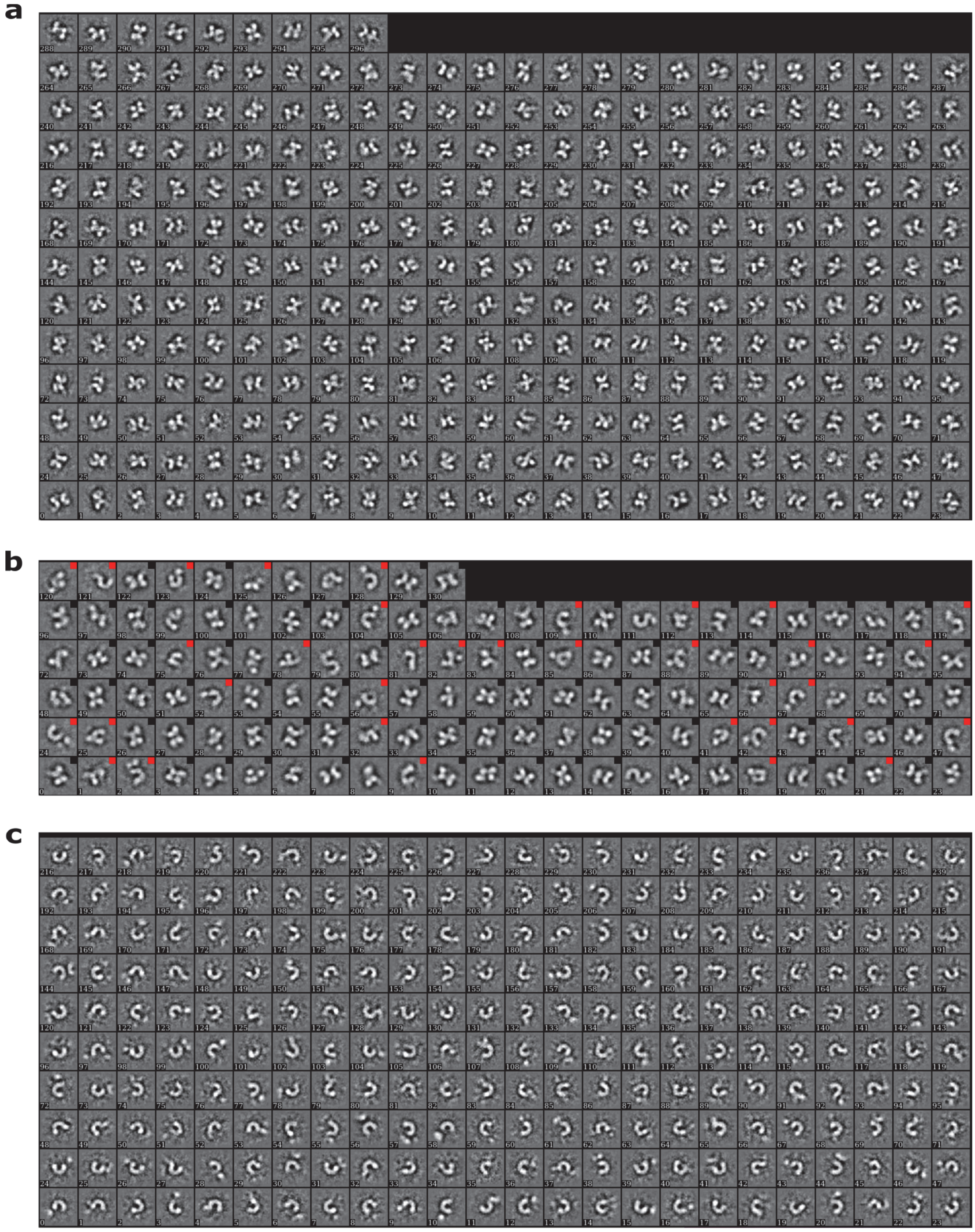

Supplementary Figure 7: Complete set of negative stain EM 2D class averages of (**a**) nucleotide-free CtRoco (using 11,571 particles), (**b**) GDP-bound CtRoco (using 9620 particles) and (**c**) GppNHp-bound CtRoco (using 11,164 particles) (box size: 18.2 x 18.2 nm). Within the set of GDP class averages (**b**), those classes that are also encountered in the nucleotide-free set (**a**) are labeled with a black square and those

classes encountered in the GppNHp set(**c**) with a red square. Some classes seem to be unique for GDP-bound CtRoco.

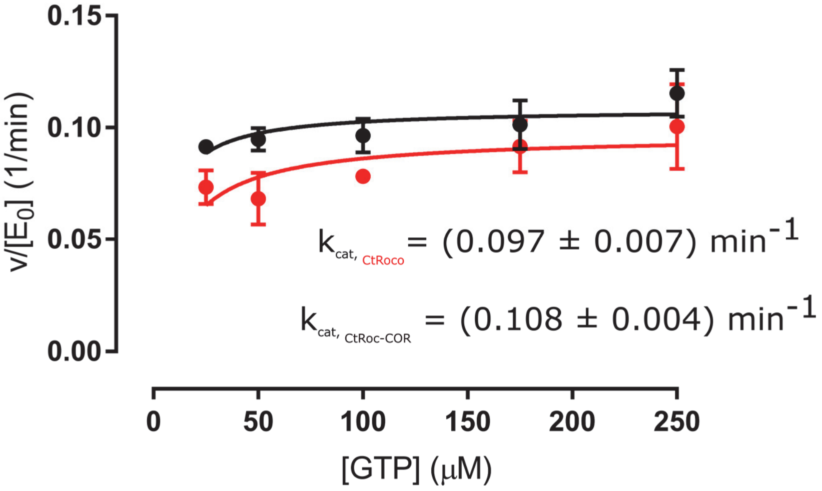

Supplementary Figure 8: Multiple turnover steady-state kinetics of CtRoco (red) and CtRoc-COR (black) at saturating substrate conditions. Fitting on the Michaelis-Menten equation gives the turnover number (k_{cat}) ± s.e. Note that the K_M value is too low to allow accurate determination. Each data point is the average (± s.d.) of 3 independent measurements.

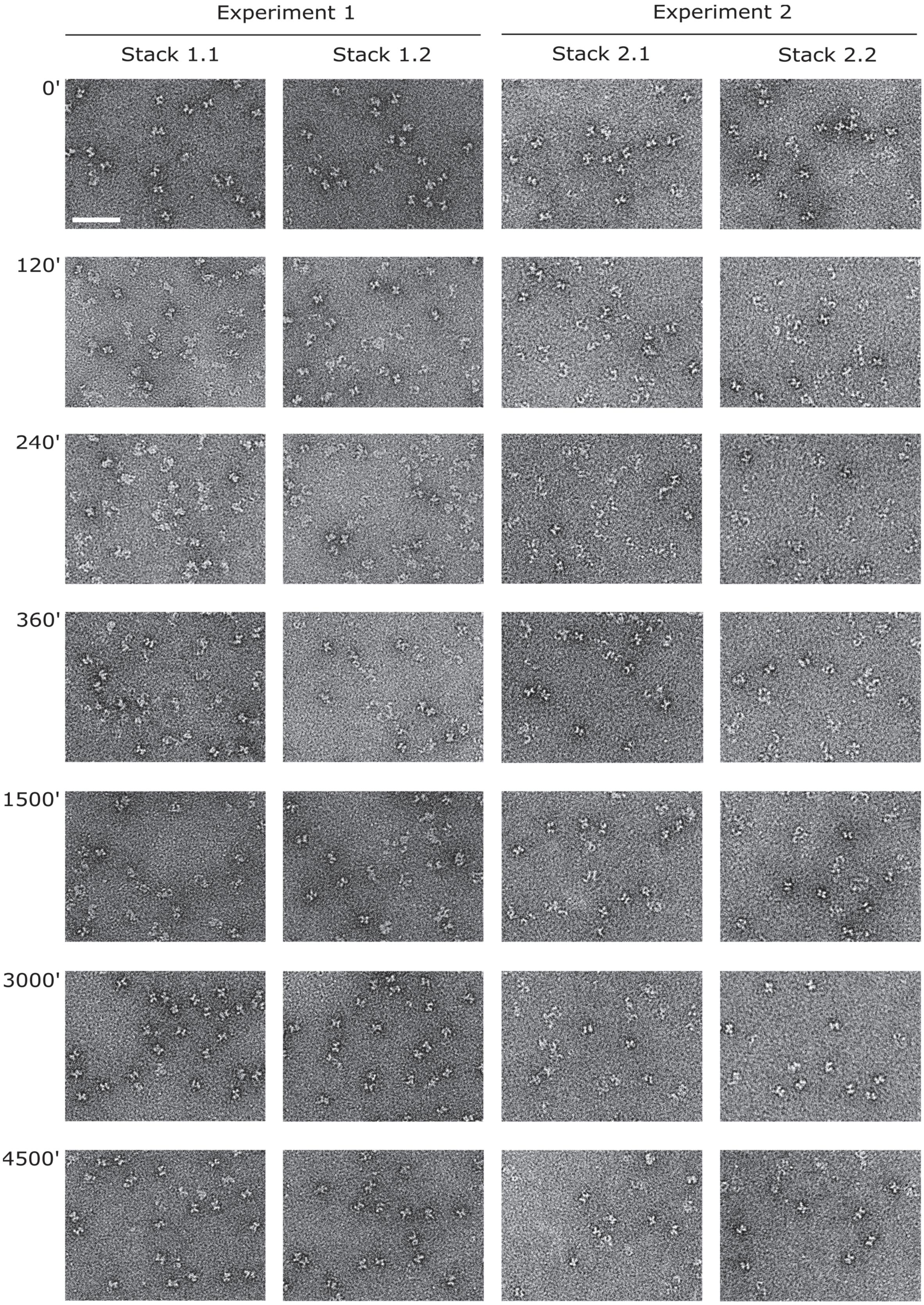
Experiment 1
Experiment 2
Stack 1.1
Stack 1.2
Stack 2.1
Stack 2.2
0'
120'
240'
360'
1500'
3000'
4500'

Supplementary Figure 9: Two repeats of a single GTP turnover experiment with CtRoco followed by time resolved EM. 1 µM CtRoco was mixed with 1 µM GTP, samples were taken at the indicated time points and applied on a glow discharged carbon-coated copper grid. Two representative images for each repeat (Experiment 1 and 2) at all time points are shown (scale bar: 50 nm).

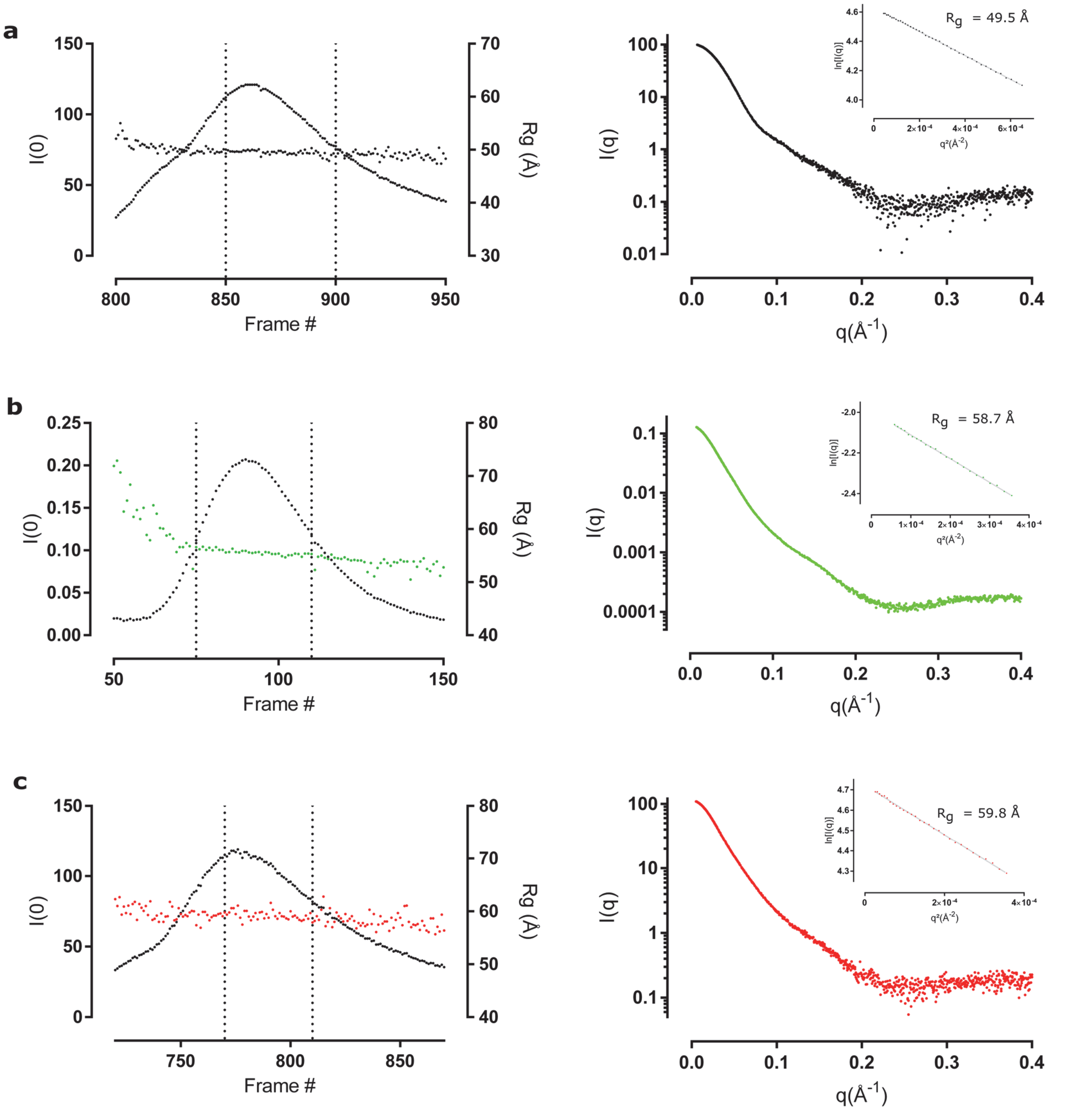

Supplementary Figure 10: (**a-c**) SEC-SAXS data of CtRoco L487A in nucleotide-free state (**a**, black), bound to GDP (**b**, green) and bound to GppNHp (**c**, red). The chromatograms of the inline size-exclusion chromatography together with the forward scattering (I(0)) and radius of gyration (R_g) obtained from collection of SAXS data on each frame are shown in the left panel. Frames within the dashed lines were selected for averaging. The corresponding final SAXS curves are shown on the right with their Guinier plots as an inset.

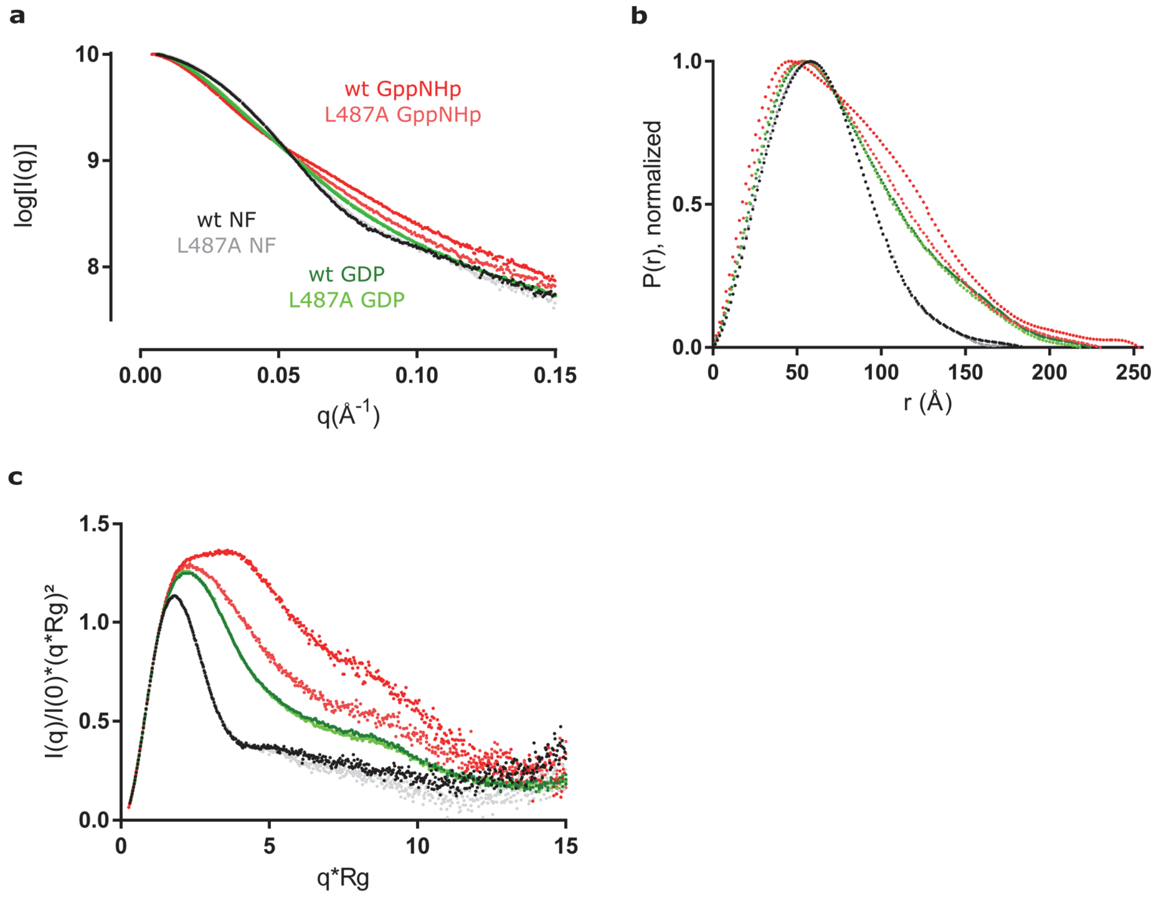

Supplementary Figure 11: Comparison of SAXS data of CtRoco wt and the CtRoco L487A mutant in nucleotide-free (NF) state or bound to GDP or GppNHp. (**a**) Scattering curves. (**b**) Normalized distance distribution functions (P(r)). (**c**) Dimensionless Kratky plots.

Supplementary Table 1: Experimental and modelling parameters of the CtRoc-COR SAXS analysis.

**Data-collection parameters:**	CtRoc-COR **NF**	CtRoc-COR **GppNHp**	CtRoc-COR **GDP**
Beamline:	SWING, SOLEIL, Gif-sur-Yvette	SWING, SOLEIL, Gif-sur-Yvette	SWING, SOLEIL, Gif-sur-Yvette
Wavelength, (Å):	1.03	1.03	1.03
q range, ($Å^{-1}$):	0.006 - 0.613	0.006 - 0.613	0.006 - 0.613
Concentration (mg/ml) (mode):	8 (HPLC)	8 (HPLC)	8 (HPLC)
Temperature, (K):	298.15	298.15	298.15
**Structural parameters:**			
I(0) (cm^{-1}) (from Guinier):	0.09484 ± 0.00004	0.06924 ± 0.00005	0.08048 ± 0.00005
R_g (Å) (from Guinier):	34.1 ± 0.03	33.64 ± 0.01	34.47 ± 0.02
I(0) (cm^{-1}) [from P(r)]:	0.09434 ± 0.00003	0.06889 ± 0.00003	0.07925 ± 0.00004
R_g (Å) [from P(r)]:	33.62 ± 0.02	33.31 ± 0.02	33.63 ± 0.02
D_{max} (Å):	100	105	100
Porod volume estimate, V_p ($Å^3$):	210780	153690	197180
**Molecular-mass determination:**			
Molecular mass MM (kDa) from Porod volume (V_p/1.7):	124	90	116
Calculated MM (kDa) from sequence (monomer):	64.9	64.9	64.9
**Modeling parameters:**			
Shape reconstruction	DAMMIN	/	/
Symmetry	P2	/	/
# of models averaged	19	/	/
DAMAVER NSD (var)	0.744 ± 0.139	/	/
**Software employed:**			
Data evaluation:	PRIMUS, GNOM	PRIMUS, GNOM	PRIMUS, GNOM
Computation of model intensities:	CRYSOL	CRYSOL	CRYSOL
Three-dimensional graphics representations:	UCSF Chimera, Pymol	UCSF Chimera, Pymol	UCSF Chimera, Pymol

Supplementary Table 2: Experimental and modelling parameters of the CtRoco SAXS analysis.

**Data-collection parameters:**	CtRoco **NF**	CtRoco **GppNHp**	CtRoco **GDP**
Beamline:	BM29, ESRF, Grenoble	BM29, ESRF, Grenoble	SWING, SOLEIL, Gif-sur-Yvette
Wavelength, (Å):	0.99	0.99	1.03
q range, ($Å^{-1}$):	0.032 - 0.498	0.032 - 0.498	0.006 - 0.553
Concentration (mg/ml) (mode):	8 (HPLC)	8 (HPLC)	8 (HPLC)
Temperature, (K):	298.15	298.15	298.15
**Structural parameters:**			
I(0) (cm^{-1}) (from Guinier):	125.13 ± 0.06	69.38 ± 0.05	0.16538 ± 0.00003
R_g (Å) (from Guinier):	49.8 ± 0.04	62.2 ± 0.08	59.0 ± 0.01
I(0) (cm^{-1}) [from P(r)]:	125.4 ± 0.06	70.24 ± 0.05	0.1675 ± 0.00002
R_g (Å) [from P(r)]:	50.4 ± 0.04	65.5 ± 0.1	61.8 ± 0.02
D_{max} (Å):	184	253	224
Porod volume estimate, V_p ($Å^3$):	440410	354480	491020
**Molecular-mass determination:**			
Molecular mass MM (kDa) from Porod volume (V_p/1.7):	259	209	289
Calculated MM (kDa) from sequence (monomer):	127.1	127.1	127.1
**Modeling parameters:**			
Shape reconstruction	DAMMIN	/	/
Symmetry	P2	/	/
# of models averaged	20	/	/
DAMAVER NSD (var)	1.047 ± 0.155	/	/
**Software employed:**			
Data evaluation:	PRIMUS, GNOM	PRIMUS, GNOM	PRIMUS, GNOM

Supplementary Table 3: Experimental and modelling parameters of the CtRoco L487A SAXS analysis.

**Data-collection parameters:**	CtRoco L487A **NF**	CtRoco L487A **GppNHp**	CtRoco L487A **GDP**
Beamline:	BM29, ESRF, Grenoble	BM29, ESRF, Grenoble	SWING, SOLEIL, Gif-sur-Yvette
Wavelength, (Å):	0.99	0.99	1.03
q range, (Å^{-1}):	0.032 - 0.498	0.032 - 0.498	0.006 - 0.553
Concentration (mg/ml) (mode):	8 (HPLC)	8 (HPLC)	8 (HPLC)
Temperature, (K):	298.15	298.15	298.15
**Structural parameters:**			
I(0) (cm^{-1}) (from Guinier):	102.51 ± 0.05	111.9 ± 0.1	0.13550 ± 0.00005
R_g (Å) (from Guinier):	49.5 ± 0.04	59.8 ± 0.1	58.7 ± 0.04
I(0) (cm^{-1}) [from P(r)]:	102.7 ± 0.05	112.7 ± 0.09	0.1363 ± 0.00003
R_g (Å) [from P(r)]:	49.9 ± 0.05	62.0 ± 0.08	60.40 ± 0.03
D_{max} (Å):	184	230	218
Porod volume estimate, V_p (Å^3):	446860	436030	469210
**Molecular-mass determination:**			
Molecular mass MM (kDa) from Porod volume (V_p/1.7):	263	256	276
Calculated MM (kDa) from sequence (monomer):	127.1	127.1	127.1
**Software employed:**			
Data evaluation:	PRIMUS, GNOM	PRIMUS, GNOM	PRIMUS, GNOM
